# Editorial: Recent advances in pediatric neuroradiology

**DOI:** 10.3389/fradi.2025.1632995

**Published:** 2025-06-03

**Authors:** Sahar Ahmad, Yunzhi Huang, Ye Wu

**Affiliations:** ^1^ Department of Radiology, The University of North Carolina at Chapel Hill, Chapel Hill, NC, United States; ^2^ Biomedical Research Imaging Center (BRIC), The University of North Carolina at Chapel Hill, Chapel Hill, NC, United States; ^3^ School of Artificial Intelligence and Future Technology, Nanjing University of Information Science and Technology, Nanjing, China; ^4^ School of Computer Science and Technology, Nanjing University of Science and Technology, Nanjing, China

**Keywords:** pediatric, neuroimaging, disease, radiology, brain development

**Editorial on the Research Topic**
Recent advances in pediatric neuroradiology

Pediatric neuroradiology is crucial for identifying abnormalities, tracking disease course, and planning treatment and assessing its efficacy in children and adolescents. The advancements in imaging techniques [magnetic resonance imaging (MRI), computed tomography (CT), and ultrasound (US)] and the latest integration of artificial intelligence (AI) have dramatically improved the pediatric neuroradiology field in both research and clinical practice. This Research Topic brings together novel studies that advance our current knowledge on pediatric imaging techniques, diagnostic procedures, and early brain development.

One of the key challenges in studying neonatal brain architecture is that during early postnatal period, brain tissue contrast and structural morphology (size, shape, and complexity) change dynamically. Since ultra-high field MRI at 7T effectively improves signal-to-noise ratio (SNR) and tissue contrast in adult brains, it appears to be a viable solution for neonatal brains. However, safety issues and regulatory constraints preclude 7T MRI acquisition from neonates. To account for this, Bridgen et al., proposed a safe 7T imaging protocol for neonates and demonstrated its improved performance for neonatal brain imaging.

Early personalized intervention remains a cornerstone in pediatric care and thus requires accurate disease diagnosis. Congenital hydrocephalus is one of the most prevalent neurological conditions in fetuses and neonates, and its incidence has increased in lower and middle-income countries (LMIC). MRI is a powerful diagnostic tool for this condition; however, it has limited availability in LMIC. An alternative is a portable ultra-low field MRI at 0.064 T, allowing bedside imaging. The application of this imaging technique was presented in a case report by Groteklaes et al., to diagnose pathologies in neonates born with congenital hydrocephalus. Another study (Sidorenko et al.) developed a predictive model based on measured clinical parameters and mathematically computed cerebral blood flow to stratify preterm infants at increased risk of developing intraventricular hemorrhage.

By capturing precise structural changes, neuroimaging biomarkers facilitate the detection of atypicalities early on. Ciceri et al., investigated the fetal brain gyrification process by quantifying different shape descriptors of cortical surfaces and predicted gestational age as a means to monitor normal fetal brain growth and identify deviations manifested in ill-formed cortical plate due to several pathologies such as spina bifida. Trimarco et al. studied the growth of thalamic volumes in very preterm infants and the neurodevelopmental outcomes at 2 years of age. Segmentation plays a key role in getting regional predictive markers. To this end, Xi et al., developed a deep learning segmentation model for CT scans of the 2-year-olds and analyzed region-specific volumes. In addition to macrostructural properties of the brain, microstructural properties computed from diffusion MRI are also important. Verschuur et al., assessed the feasibility of constrained spherical deconvolution (CSD) in reconstructing crossing fibers from neonatal data and also analyzed the impact of spatial and angular resolution, as well as processing parameters, on tractography and quantitative metrics.

This Research Topic also includes two review articles: (i) Shen and Zhou, discussed advancements in studying attention-deficit/hyperactivity disorder (ADHD), including insights from neuroimaging, and (ii) Zhang et al., presented a pediatric case on myelin oligodendrocyte antibody-associated transverse myelitis (MOG-TM) and discussed their findings in the context of previous observations.

To summarize, this special issue covers a breadth of topics in pediatric neuroradiology that will inform the readers about the latest advancements in neuroimaging, image analysis, brain development, and disease diagnosis.

